# Biomimetic Construction of *Enteromorpha prolifera*-Based Composite Membranes for Synergistic Purification of Fluoride Ions, Bacteria, and Dye with High Sustainability

**DOI:** 10.3390/ma18102356

**Published:** 2025-05-19

**Authors:** Wanying Li, Yu Lei, Xiaoxuan Fan, Gang Wei, Lei Guo

**Affiliations:** 1 Research Center for High-Value Utilization of Waste Biomass, Institute of Biomedical Engineering, College of Life Science, Qingdao University, Qingdao 266071, China; liwanying0718@163.com (W.L.); llleiyu0805@163.com (Y.L.); 15065645330@163.com (X.F.); 2 College of Chemistry and Chemical Engineering, Qingdao University, Qingdao 266071, China

**Keywords:** *Enteromorpha prolifera*, biomineralization, composites, functional membrane, water purification

## Abstract

As an essential trace element in the human body, fluoride is beneficial in appropriate amounts, but excessive intake can cause serious harm. Therefore, addressing the global water pollution caused by fluoride is an urgent issue. In this study, a functional composite membrane is successfully prepared using *Enteromorpha prolifera* (EP) as the raw material, cinnamaldehyde (CIN) as a functional modifier, and EP-bioinduced ZrO_2_ nanoparticles (NPs) as the loading material via biomimetic mineralization technology. The experimental results demonstrate that the composite membrane removes fluoride ions (F^−^) with an efficiency of over 99.9% within the concentration range of 100–400 mg/L. This excellent F^−^ removal performance is attributed to the ability of the hydroxyl groups on the surface of ZrO_2_ to exchange and bind with F^−^. The formed CIN/EP-ZrO_2_ composite membrane also reveals significant antibacterial activity against *E. coli*. In addition, the adsorption rate for methylene blue at the concentration of 5–300 mg/L reaches 99.99%, which is due to the synergistic interaction of functional groups such as hydroxyl (-OH), carboxyl (-COOH), and amino groups (-NH_2_) in EP. The overall sustainability footprint (OSF) assessment exhibits that the CIN/EP-ZrO_2_ composite membrane has comprehensive advantages, including a simple preparation process, low cost, high performance, and environmental friendliness. This study provides an innovative solution for the sustainable treatment of F^−^, bacteria, and dye pollution in water, showcasing significant potential for applications in environmental science.

## 1. Introduction

Fluorine is a naturally occurring element, typically found in the form of fluoride, which is an essential trace element for the human body. It plays a crucial role in the formation of bones and is beneficial to health within a specific concentration range [1]. The World Health Organization (WHO) recommends that the ideal fluoride concentration in drinking water should be between 0.5 and 1.5 mg/L [2]. However, an excessive intake of fluoride can lead to serious health issues, such as fluorosis, dental fluorosis, DNA damage, Alzheimer’s syndrome [3,4,5], and cancerous growths in the liver, bladder, thyroid, skin, lung, and kidney [6].

As one of the most chemically active non-metallic elements, fluorine can directly or indirectly combine with other elements to form various fluorides at room temperature. The main sources of fluoride contamination include industrial activities, such as electronics [7], ceramics, semiconductors [8], steel, electroplating, and fluorine-rich minerals like fluorite, cryolite, and fluorapatite [9]. Fluoride can enter groundwater through contact with surface water, leading to the widespread presence of fluoride ions (F^−^) in groundwater across most regions of the world. It is one of the most abundant anions in groundwater and a significant water pollutant [10]. Currently, fluoride contamination in water has become a global problem. The main defluorination methods developed to address this problem include adsorption [11], ion exchange [12], chemical precipitation [13], electrodialysis [14], and membrane filtration [7]. Among these, adsorption has been widely used due to its low cost, simplicity, and high adsorption efficiency.

One of the key points of the adsorption method is the selection of the adsorbent material. For instance, He et al. used a metal–organic framework (MOF) as the adsorbent material to prepare a Zr-MOF membrane for fluoride removal from drinking water [15]. At a fluoride concentration of 200 mg/L, the maximum adsorption capacity was 102.40 mg/g, and the highest fluoride removal rate achieved was 98%. In another study, Dhongde et al. developed a novel ZrAlCa nano-hybrid crystalline fluoride removal adsorbent using a coprecipitation method [16]. The adsorption performance of this material was systematically investigated through batch adsorption experiments, demonstrating a remarkable fluoride removal efficiency of up to 99% across both near-neutral and acidic pH ranges.

In recent years, biomass materials have attracted much attention as promising adsorbents due to their low cost and environmental friendliness [17]. These materials are considered highly advantageous for defluoridation. Zirconium (Zr) is often used to modify polymeric materials to improve their defluoridation performance, owing to its high electrical activity, low oral toxicity, and specific adsorption to fluorine [18]. For instance, Mei et al. prepared a chitosan (CS)/polyvinyl alcohol (PVA)-La-Zr composite adsorbent using CS as the raw material [19]. The adsorbent had the advantage of rapid adsorption and was able to remove over 90% of fluoride from water within just 1 min, with a maximum adsorption capacity of 30.73 mg/g. Similarly, using CS as the raw material, Zhang et al. fabricated a Zr-CS/graphene oxide (Zr-CS/GO) membrane adsorbent for fluoride removal [20], achieving a maximum adsorption capacity of 29.0588 mg/g. Li et al. also developed an effective adsorbent for fluoride removal by depositing polypyrrole onto granular peanut shell biocarbon (BC) by in situ chemical oxidative polymerization, using peanut shell as the raw material [21].

*Enteromorpha prolifera* (EP) has shown great potential for the fabrication of biomass-based composite membranes. For example, Lei et al. used seaweed residue (EP) as the raw material to prepare an activated GO/seaweed residue–zirconia composite filter membrane [22], which achieved over 99% removal efficiency for fluoride in the range of 100–400 mg/L in water. EP is rich in polysaccharides, proteins, cellulose, and lipids, which endow it with rich functional groups, such as the sulfhydryl group (-SH), carboxyl group (-COOH), sulfur atom, amino group (-NH_2_), phosphate group, and oxygen atom. These diverse functional groups could significantly improve the adsorption of pollutants through electrostatic interactions and chelation [23,24,25,26]. Additionally, GO is considered a promising antibacterial material due to its large specific surface area and the presence of rich functional groups, such as the hydroxyl group (-OH), carboxyl group (-COOH), and epoxy group [27,28].

**Scheme 1:**
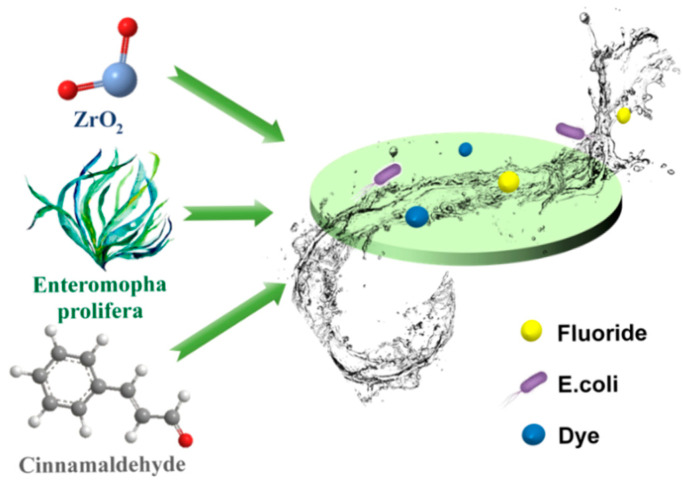
Construction of CIN/EP-ZrO_2_ composite membrane for the adsorption and removal of F^−^, *E. coli*, and dye.

In this study, cinnamaldehyde (CIN), a cost-effective and antibacterial compound, was selected as a functional modifier. CIN is a phytogenic plant compound isolated from cinnamon essential oil, known for its natural antibacterial and anti-biofilm properties. It is characterized by low water solubility [29] and exhibits biological activities, including antibacterial [30,31], anticancer [32], anti-diabetic [33], and wound-healing promotion [34]. Therefore, we take EP as the raw material and biomimetically mineralize ZrO_2_ nanoparticles on CIN-loaded EP to prepare a functional filter membrane, CIN/EP-ZrO_2_, for fluoride adsorption from water. This membrane also exhibits effective antibacterial properties and dye adsorption capabilities, as illustrated in Scheme 1. The results indicate that the CIN/EP-ZrO_2_ hybrid membrane performs excellently in fluoride adsorption, as well as antibacterial and dye removal applications. The preparation method of the biomass composite filter membrane in this study is simple, environmentally friendly, and cost effective, providing a promising solution for the removal of pollutants from water bodies.

## 2. Materials and Methods

### 2.1. Chemicals and Materials

EP was collected from the Yellow Sea of China (Qingdao). CIN, zirconium oxychloride octahydrate (ZrOCl_2_·8H_2_O, 98%), and sodium fluoride (NaF, 98%) were purchased from the Macklin Company (Shanghai, China). Na_2_SO_4_ (99%), NaOH (96%), HCl (36–38%), NaCl (99%), and methylene blue (MB) (C_16_H_18_CIN_3_S·3H_2_O) were purchased from the Sinopharm Chemical Reagent Co., Ltd. (Beijing, China). The bacterial strain *Escherichia coli* ATCC 25922 was obtained from Shanghai Macklin Biochemical Co., Ltd. (Shanghai, China). All chemicals used in this work were of analytical reagent grade, used directly, and did not require additional purification. Deionized (DI) water was generated from a Millipore Milli-Q water system (Millipore Inc., Burlington, MA, USA) by a laboratory ultra-pure water machine (resistivity = 18 MΩ·cm).

### 2.2. Preparation of CIN/EP-ZrO_2_ Composite Membrane

EP was firstly washed and dried, then crushed, and sieved through a 500-mesh screen to obtain the EP powder. Then, 1g EP powder was dispersed in DI water and heated in a 100 °C water bath for 20 min. The heated EP dispersion was pumped and filtered in a vacuum suction and filtration device to obtain the EP solid. After that, the CIN/EP mixture was obtained by dispersing 10 μL of CIN in 15 mL absolute ethanol and adding the EP solid under stirring for 24 h. Then, 0.3 g ZrOCl_2_·8H_2_O was added into the CIN/EP mixture, and the CIN/EP-ZrO_2_ mixture was obtained under stirring for 8 h. As the solution was highly acidic, the pH of the solution was adjusted to 3.5–4 by slowly adding NaOH. Finally, the CIN/EP-ZrO_2_ composite membrane was constructed after the mixture was filtered by a vacuum filtration device and washed 3 times.

### 2.3. Adsorption Experiments of F^−^

*Effect of different amounts of ZrOCl_2_·8H_2_O on F^−^ adsorption:* The CIN/EP-ZrO_2_ composite membranes were prepared by weighing 0.3, 0.6, and 0.9 g ZrOCl_2_·8H_2_O, respectively. The CIN/EP-ZrO_2_ composite membranes were used as the filters, and 5 mL F^−^ (prepared with 98% NaF) solution with a concentration of 300 mg/L was quickly filtered by a vacuum filtration device. The operating pressure was −0.1 MPa. When the filtration was completed, the filtrate was collected and the residual F^−^ concentration was determined using a UV–vis spectrophotometer, and the measurement wavelength was 620 nm.

*Adsorption performance test:* Then, 1 g EP and CIN/EP-ZrO_2_ composite membrane was used as the filter, and 5 mL F^−^ (prepared with 98% NaF) solution with concentrations of 100, 200, 300, and 400 mg/L were rapidly pumped by a vacuum filtration device, respectively. The operating pressure was −0.1 MPa. After the filtration was completed, the filtrate was collected and the residual F^−^ concentration was determined using a UV–vis spectrophotometer, and the measurement wavelength was 620 nm.

*Regeneration performance test:* The CIN/EP-ZrO_2_ composite membrane was used as a filter, and 5 mL F^−^ (prepared with 98% NaF) solution with a concentration of 300 mg/L was quickly aspirated by a vacuum filtration device. The filtered CIN/EP-ZrO_2_ composite membrane was immersed in 1% NaOH and 5% NaCl solution for 30 min, and then 5 mL F^−^ (prepared with 98% NaF) solution (300 mg/L) was filtered again. A total of 3 cycles of regeneration experiments were performed. The operating pressure was −0.1 MPa. After the filtration was completed, the filtrate was collected and the residual F^−^ concentration was determined using a UV–vis spectrophotometer, and the measurement wavelength was 620 nm.

*Dynamic adsorption test:* The prepared CIN/EP-ZrO_2_ composite membrane was dried and ground into a powder for later use. A total of 2000 mL of F^−^-containing solution with a concentration of 100 mg/L was prepared, and 0.05 g of the sample was added to the F^−^-containing solution and stirred at 20 °C. Then, 2 mL solution was centrifuged at 11,000 rpm for 5 min and the supernatant was collected. Residual F^−^ concentration was determined using a UV–vis spectrophotometer at a wavelength of 620 nm. The adsorption capacity of F^−^ is calculated by the Formula (1), and the adsorption results were fitted with the pseudo-first-order model (2) and the pseudo-second-order model (3).

(1) $$q_{t}=\frac{\left(C_{0}-C_{t}\right)V}{m}$$

where C_0_ is the concentration of F^−^ in the initial solution (mg/L); C_t_ is the concentration of F^−^ in solution (mg/L) when the contact time is t; m is the mass of adsorbent (mg); and V is the volume of solution (mL).

Two models of pseudo-first-order equation and pseudo-second-order equation were used to analyze the kinetic data of F^−^-adsorbed CIN/EP-ZrO_2_ composite membrane. The formulae of the two models were as follows:

(2) $$\text{Pseudo-first-order formula: }ln\left(q_{e}-q_{t}\right)=lnq_{e}-K_{1}t$$

(3) $$\text{Pseudo-second-order formula: }\frac{t}{q_{t}}=\frac{t}{q_{e}}+\frac{1}{K_{2}q_{e}^{2}}$$

where q_e_ is the adsorption amount (mg/g) when the adsorption reaches equilibrium; q_t_ is the adsorption amount (mg/g) when the contact time between the adsorbent and the solution is t; and K_1_ and K_2_ are the adsorption rate constants (g/mg min).

*Adsorption isotherm test:* The prepared CIN/EP-ZrO_2_ composite membrane was dried and ground into a powder for later use. Then, 200 mL of F^−^-containing solutions with concentrations of 5, 15, 25, 50, 75, 100, 150, and 200 mg/L were prepared, respectively. A total of 0.01 g of the sample was added into the F^−^-containing solutions at 20 °C under stirring. Then, 2 mL solution was centrifuged at 11,000 rpm for 5 min to collect the supernatant. Residual F^−^ concentration was determined using a UV–vis spectrophotometer at a wavelength of 620 nm. The equilibrium adsorption quantity calculated by Formula (4) as follows:

(4) $$q_{e}=\frac{\left(C_{0}-C_{e}\right)V}{m}$$

where C_e_ is the concentration (mg/L) of F^−^ in the solution when the adsorption reaches equilibrium.

The adsorption isotherm was calculated by two adsorption models, Langmuir and Freundlich, using Formulae (5) and (6) as follows:

(5) $$\frac{C_{e}}{q_{e}}=\frac{1}{q_{m}K_{L}}+\frac{C_{e}}{q_{m}}$$

(6) $$lnq_{e}=lnK_{F}+\frac{1}{n}lnC_{e}$$

where q_e_ represents the adsorption amount at equilibrium (mg/g); q_m_ represents saturated adsorption capacity (mg/g); K_L_ is a constant related to adsorption capacity and energy (L/mg); and K_F_ and n are constants related to adsorption capacity and adsorption strength.

### 2.4. Antibacterial Testing

*E. coli* was inoculated in LB medium and incubated at 37 °C and 150 r/min for 12 h to prepare bacterial suspension, which was then diluted to 1.5 × 10^5^ CFU/mL with sterile water for later use. The sterilized LB nutrient agar was heated to melting point, poured into Petri dishes when cooled to 50 °C, and allowed to air dry.

*Effect of different CIN dosage on the antibacterial property of CIN/EP-ZrO_2_ composite membrane:* The CIN/EP-ZrO_2_ composite membranes were prepared by weighing 5, 10, and 30 μL of CIN, respectively. Under sterile conditions, the prepared CIN/EP-ZrO_2_ composite membranes were used as the filters, and 5 mL of 1.5 × 10^5^ CFU/mL bacterial suspension was treated by rapid vacuum filtration. The operating pressure was set to −0.1 MPa, and the filtrate was collected after the filtration process was completed.

*Antibacterial properties of EP, CIN/EP, and CIN/EP-ZrO_2_ composite membrane:* A total of 1 g EP, CIN/EP, and CIN/EP-ZrO_2_ composite membrane were used as the filters, respectively. Under sterile conditions, a vacuum filtration device was used to perform both direct rapid filtration and stirred rapid filtration of 5 mL of bacterial suspension with a concentration of 1.5 × 10⁵ CFU/mL. The operating pressure was −0.1 MPa. The filtrate was collected after the filtration was completed.

After dilution, 100 μL of bacterial suspension and 100 μL of filtrate were each evenly spread on agar plates. The plates were then incubated at 37 °C for 12 h in a constant-temperature air bath incubator, after which colonies were counted and observed.

### 2.5. Adsorption Experiment of MB

Using the CIN/EP-ZrO_2_ composite membrane as a filter, 5 mL MB solution at concentrations of 1, 5, 20, 50, 100, and 300 mg/L were rapidly filtered by vacuum filtration device, respectively. The operating pressure was set to −0.1 MPa. After filtration, the filtrate was collected and the remaining MB concentration was determined using a microplate reader.

### 2.6. Sustainability Analysis

The overall sustainability footprint (OSF) method was used for multivariate analysis of the CIN/EP-ZrO_2_ composite membranes, and the results were compared with those of biomass films reported in other studies. Based on the three principles of sustainable development (ecological sustainability, economic sustainability, and social sustainability), the following eight factors were selected for the sustainability analysis: preparation simplicity, public acceptability, environmental friendliness, recyclability, degradability, removal efficiency, cost, and waste utilization. By rating the impact as low, medium, and high as a sustainability criterion, the OSF values were calculated using the following formula [35]:

(7) $$OSF=100\%\times \sum_{j=1}^{8}{\left(\frac{i}{3}\right)}_{j}\times \frac{1}{8}$$

where j represents the eight sustainability factors and *i* is their corresponding scores. Assessment was performed on a three-level basis, with low (L, *i* = 1), medium (M, *i* = 2), and high (H, *i* = 3) level scores. Special attention should be paid to the analytical evaluation of “cost” where L stands for high cost and H for low cost.

### 2.7. Characterization Techniques

Metallographic microscopy was used to characterize the morphology of EP, CIN, and CIN/EP. The morphology and structure of EP, CIN/EP, and CIN/EP-ZrO_2_ were observed by scanning electron microscopy (SEM, Regulus 8100, Hitachi, Tokyo, Japan). The chemical structures of the composite membranes were analyzed using a Fourier transform infrared spectrometer (FT-IR, Nicolet iS 50, Nicolet Instrument, Madison, WI, USA). The composition of the CIN/EP-ZrO_2_ composite membrane was analyzed by X-ray diffraction (XRD) analysis carried out on an X-ray diffractometer.

## 3. Results and Discussion

### 3.1. Preparation and Characterizations of the CIN/EP-ZrO_2_ Composite Membranes

**Figure 1:**
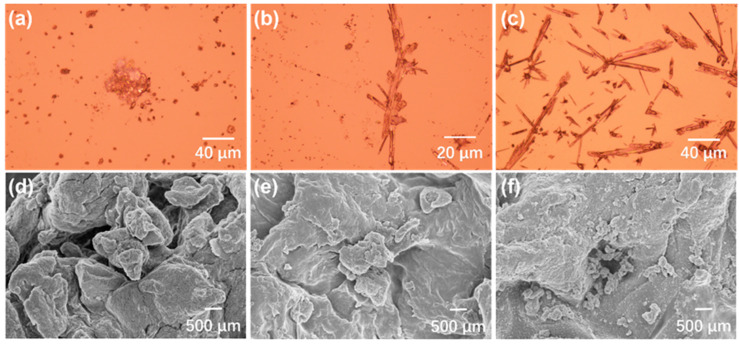
(**a**–**c**) Metallographic microscope characterization of materials: (**a**) EP, (**b**) CIN, and (**c**) CIN/EP. (**d**–**f**) SEM images of materials: (**d**) EP, (**e**) CIN/EP, and (**f**) CIN/EP-ZrO_2_.

The morphologies of EP, CIN, and CIN/EP were characterized using a metallographic microscope. Figure 1a shows EP as aggregates of varying sizes, and Figure 1b presents the morphology of CIN, while Figure 1c depicts the structure of the CIN/EP mixture after the modification, clearly indicating EP has been composited with CIN, thus confirming the successful modification of EP by CIN.

Subsequently, the different stages of the preparation process of the CIN/EP-ZrO_2_ composite membrane were observed and analyzed in detail by SEM. Figure 1d shows the morphology of EP. Figure 1e shows the topography of the CIN/EP mixture, which shows a smoother morphology than EP alone. Figure 1f demonstrates the morphology of the CIN/EP-ZrO_2_ composite membrane after adding ZrOCl_2_·8H_2_O, in which the surface appears notably rougher. EP contains a wealth of charged functional groups, such as -OH, -COOH, and -NH_2_, which not only lay the groundwork for subsequent adsorption but also offer numerous active sites for the adsorption of positively charged Zr^4+^. Through covalent bonding and electrostatic interactions, CIN/EP and ZrO_2_ were combined, creating suitable conditions for the in situ biomimetic mineralization of ZrO_2_ NPs. Previous studies have proved that the formation of ZrO_2_ NPs can be promoted by adjusting the pH of the solution to between 3.5 and 4.0 [36,37].

**Figure 2:**
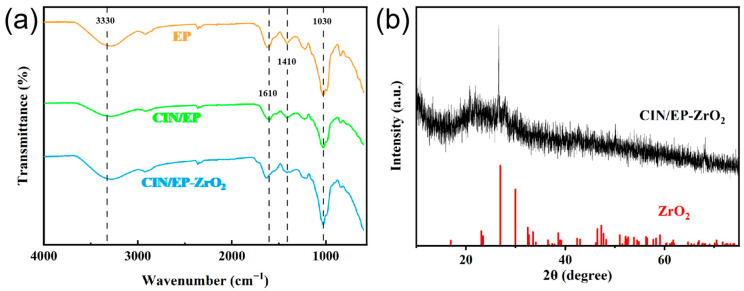
(**a**) FT-IR characterizations of EP, CIN/EP, and CIN/EP-ZrO_2_. (**b**) XRD spectrum of the CIN/EP-ZrO_2_ membrane.

Figure 2a shows the FTIR spectra of EP, CIN/EP, and CIN/EP-ZrO_2_. In the spectrum of EP, characteristic peaks are located at 1030, 1410, 1610, and 3330 cm^−1^. The strong peak at 1030 cm^−1^ is assigned to the stretching vibrations of C-O-C glycosidic bonds and C-O in polysaccharides, confirming the polysaccharide structure of EP. The absorption peaks at 1410 and 1610 cm^−1^ likely correspond to the N-H bending and the C-N stretching vibrations, or the C=O and O-H bending vibrations [22]. The peak at 1610 cm^−1^ in CIN/EP also indicates the C=C stretching vibrations from CIN’s aromatic structure, suggesting its retention during the composite process [38]. In the spectrum of CIN/EP-ZrO_2_, the characteristic peak at 1060 cm^−1^ shows no significant shift, indicating no chemical cleavage of CIN’s aromatic structure, but the physical adsorption of CIN onto the EP surface. A broad peak at 3330 cm^−1^ is ascribed to the O-H and N-H stretching vibrations in polysaccharides and proteins of EP. 

The formation of ZrO_2_ NPs on CIN/EP was further confirmed by XRD. The XRD pattern of CIN/EP-ZrO_2_ (Figure 2b) was compared with the standard XRD pattern of ZrO_2_, and the characteristic peak of standard ZrO_2_ appears in the XRD pattern of CIN/EP-ZrO_2_ after the biomimetic mineralization. Therefore, the successful biomimetic synthesis of ZrO_2_ NPs can be proved.

### 3.2. F^−^ Adsorption of the CIN/EP-ZrO_2_ Composite Membranes

**Figure 3:**
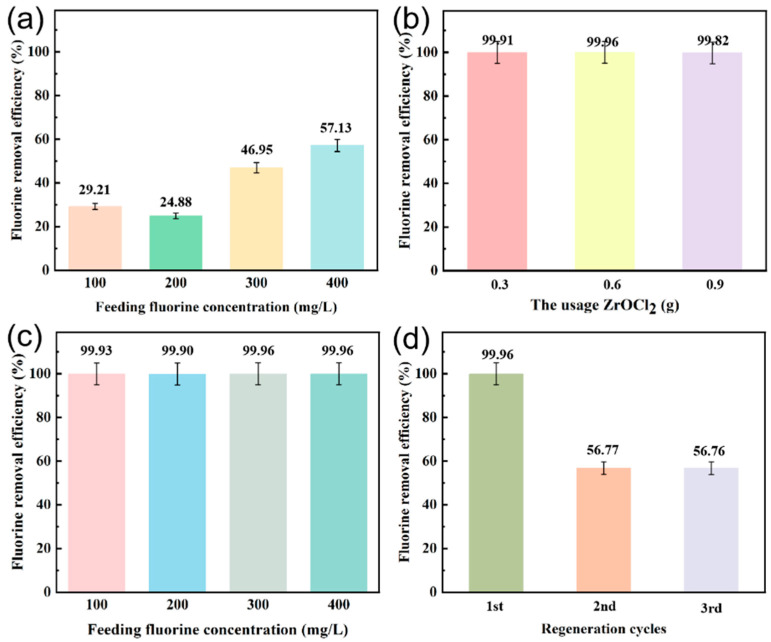
(**a**) Removal efficiency of EP for different concentrations of F^−^, (**b**) removal efficiency of the CIN/EP-ZrO_2_ composite membranes with different ZrO_2_ loading (F^−^ concentration is 300 mg/L), (**c**) removal efficiency of the CIN/EP-ZrO_2_ composite membrane for different concentrations of F^−^, (**d**) regeneration test of the CIN/EP-ZrO_2_ composite membrane on F^−^ removal efficiency.

We then investigated the F^−^ adsorption effect of EP alone, and the experimental results are shown in Figure 3a. When the initial concentration of the F^−^ solution was 100 and 200 mg/L, the removal efficiency was 29.21% and 24.88%, respectively. When the initial concentration was increased to 300 mg/L, the removal efficiency was also increased to 46.95%. When the initial concentration was further raised to 400 mg/L, the removal rate reached 57.13%. To show the advantages of the CIN/EP-ZrO_2_ composite membrane for F^−^ adsorption, the effect of different dosage of ZrOCl_2_·8H_2_O on the F^−^ removal performance was studied (Figure 3b). The CIN/EP-ZrO_2_ composite membranes were prepared by adding 0.3, 0.6, and 0.9 g ZrOCl_2_·8H_2_O as filters, respectively. When a concentration of 300 mg/L F^−^ solution was applied to all the membranes, the removal efficiency was 99.91%, 99.96%, and 99.82%, respectively. Therefore, the dosage of Zr precursor has no clear effect on the removal efficiency of the formed composite membranes.

Considering the cost factor, 0.3 g ZrOCl_2_·8H_2_O was utilized to prepare the CIN/EP-ZrO_2_ composite membrane in the subsequent experiments. We further investigated the adsorption performance of the CIN/EP-ZrO_2_ composite membrane on different concentrations of F^−^. As shown in Figure 3c, when the initial concentration of the F^−^ solution was 100 mg/L, the removal rate was 99.93%. When the initial concentration was changed to 200 mg/L, the removal efficiency reached 99.90%. When the initial concentration was further increased to 300 and 400 mg/L, the removal rates were nearly 99.96%. Finally, we investigated the regeneration performance of the CIN/EP-ZrO_2_ composite membrane (Figure 3d). After removing the F^−^ solution of a concentration of 300 mg/L, the removal efficiency of the membrane after the first, second, and third removal was 99.96%, 56.77%, and 56.76%, respectively. These experimental results indicate that the CIN/EP-ZrO_2_ composite film has excellent F^−^ adsorption properties.

**Figure 4:**
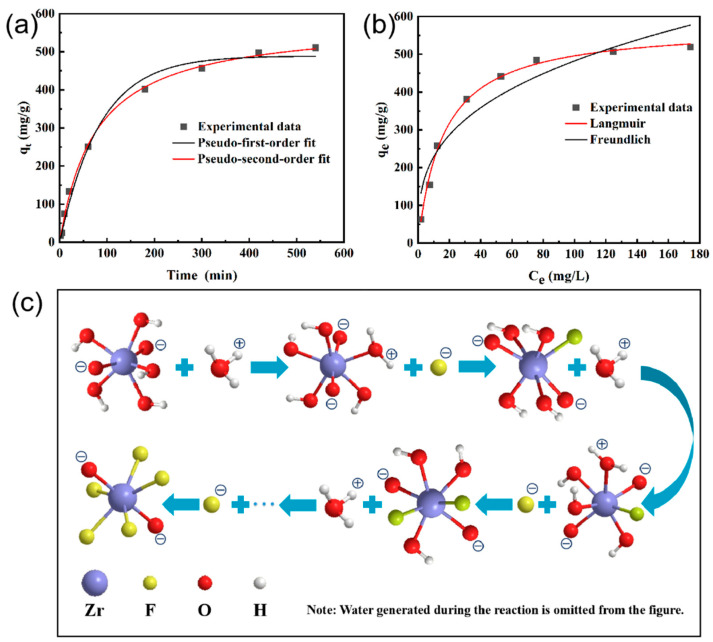
(**a**) Pseudo-first-order and pseudo-second-order adsorption kinetics of F^−^ of the CIN/EP-ZrO_2_ composite membranes. (**b**) Simulation of isothermal adsorption of F^−^ by the CIN/EP-ZrO_2_ composite membrane. (**c**) Adsorption mechanism of F^−^.

We further investigated the adsorption kinetics and isotherms of the CIN/EP-ZrO_2_ composite membrane. Figure 4a shows the pseudo-first-order and pseudo-second-order kinetic fitting curves for the membrane. From Figure 4a, it can be seen that the adsorption capacity increased rapidly in the initial 0–100 min. This is because there were numerous active sites on the membrane surface available to react with F^−^. As these sites became occupied, the adsorption rate gradually decreased. After 300 min, the adsorption process essentially reached equilibrium. Table 1 lists the obtained kinetic parameters. The pseudo-first-order and pseudo-second-order kinetics represent the physical and chemical adsorption processes, respectively. The data in the table indicate that both models can describe the adsorption process due to their high R^2^ values. However, the pseudo-second-order model has a higher linear correlation coefficient (R^2^ > 0.99), and the adsorption capacity (Qe, cal) calculated from this model is closer to the actual measured values. Therefore, chemical adsorption is likely the primary rate-limiting step for F^−^ adsorption by the CIN/EP-ZrO_2_ composite membrane.

We used the Langmuir and Freundlich adsorption isotherm models to simulate the CIN/EP-ZrO_2_ composite membrane’s adsorption towards F^−^. Figure 4b shows the adsorption isotherm of F^−^ onto the CIN/EP-ZrO_2_ composite membrane. Table 2 presents the isotherm fitting results, indicating a maximum adsorption capacity of 577.1421 mg/g for F^−^. Given the Langmuir model’s higher R^2^, it better fits the adsorption process of F^−^ onto the CIN/EP-ZrO_2_ composite membrane.

Here, we selected the same type of F^−^ adsorbent material for comparison. The results in Table 3 indicate that the adsorption capacity of the CIN/EP-ZrO_2_ composite membrane for F^−^ reached 577.1421 mg/g, which is better than many other F^−^ adsorption materials reported. In addition, EP, the raw material we selected, is itself an algae that causes marine disasters, which has the advantages of a low cost and environmental protection.

The F^−^ removal mechanism of the CIN/EP-ZrO_2_ composite membrane was then explored. As shown in Figure 4c Zr has high electroactivity, and Zr and its metal oxides can strongly bind with F^−^ [20,39,47]. The -OH groups on the surface of ZrO_2_ NPs are crucial for F^−^ adsorption. During the adsorption, the charged ZrO_2_ NPs interacted electrostatically with F^−^ ions, and F^−^ replaced the surface -OH groups to form zirconium–fluoride complexes like ZrO_2_F_5_ and ZrO_3_F_4_ [22,48,49].

### 3.3. Antibacterial Properties of the CIN/EP-ZrO_2_ Composite Membranes

**Figure 5:**
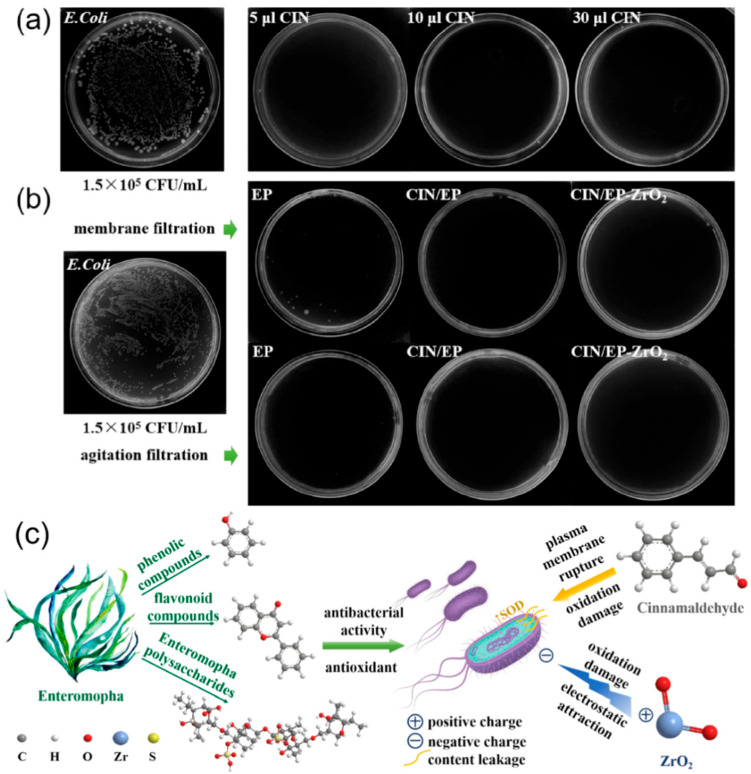
The antimicrobial ability of the CIN/EP-ZrO_2_ composite membranes: (**a**) antimicrobial effects of different amounts of CIN, (**b**) antimicrobial effects of stirring and membrane filtration of EP, CIN/EP, and CIN/EP-ZrO_2_, and (**c**) antibacterial mechanism.

We investigated the effect of the CIN dosage on the antibacterial property of the CIN/EP-ZrO_2_ composite membranes, by adding 5, 10, and 30 μL of CIN to prepare the CIN/EP-ZrO_2_ composite membranes, respectively. The experimental results are shown in Figure 5a. Compared with a 1.5 × 10^5^ CFU/mL *E. coli* solution as control, the CIN/EP-ZrO_2_ composite membranes prepared with three different CIN dosages all exhibited excellent antibacterial effects.

The CIN/EP-ZrO_2_ composite membrane was prepared using 10 μL CIN in the subsequent experiments. In order to further investigate the antibacterial properties of the CIN/EP-ZrO_2_ composite membrane, we performed direct and stirred rapid filtration experiments on 1 g EP, CIN/EP, and CIN/EP-ZrO_2_ composite membranes, respectively. As shown in Figure 5b, compared with 1.5 × 10^5^ CFU/mL *E. coli* bacterial solution, EP had a certain antibacterial effect on *E. coli*, and a small part of the bacterial solution remained. The CIN/EP mixture and CIN/EP-ZrO_2_ composite membrane demonstrated an enhanced antibacterial effect in both direct rapid filtration and stirred rapid filtration, and the sterile liquid was left. The results prove that the CIN/EP-ZrO_2_ composite membrane exhibited excellent antibacterial properties.

We then studied the antibacterial mechanism of the CIN/EP-ZrO_2_ composite membrane (Figure 5c). EP contains polysaccharides, flavonoids, and phenolic compounds, which all have antioxidant and antibacterial properties [50,51]. CIN affects cell morphology, causing membrane rupture and content leakage [52]. It also induces oxidative damage to the cell membrane of *E. coli*, increasing total SOD activity [53]. Moreover, the positive charges on the CIN/EP-ZrO_2_ surface attract the negatively charged *E. coli*, leading to its oxidation and cell death [54]. We suggest that the synergistic effects of EP, CIN, and ZrO_2_ give the composite membrane excellent antibacterial properties.

### 3.4. Dye Adsorption Properties of the CIN/EP-ZrO_2_ Composite Membranes

**Figure 6:**
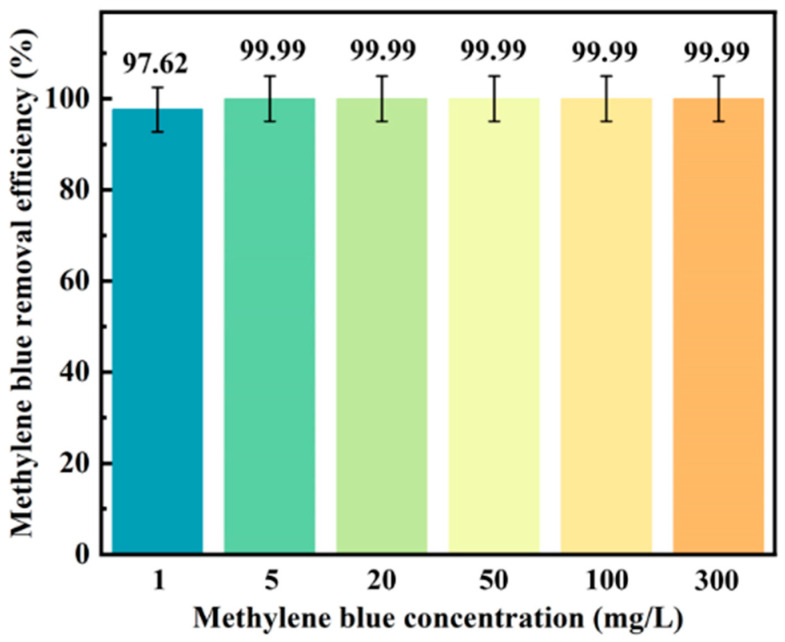
Adsorption of methylene blue at different concentrations of the CIN/EP-ZrO_2_ composite membranes.

We further investigated the dye adsorption properties of the CIN/EP-ZrO_2_ composite membranes. As shown in Figure 6, when the initial concentration of the MB solution was 1 mg/L, the removal rate reached 97.62%. When the initial concentrations of MB solution were 5, 20, 50, 100, and 300 mg/L, the removal efficiency could reach 99.99% in all cases. The results indicate that the CIN/EP-ZrO_2_ composite membranes have excellent dye adsorption performance.

Previous studies have shown that EP contains abundant -COOH, -NH_2_, and -OH groups, which were crucial for the dye adsorption. These groups interact with MB molecules, particularly via hydrogen bonds formed between -OH and the S, N, Cl, and O atoms in MB’s structure [55], which enabled the CIN/EP-ZrO_2_ composite membrane to exhibit excellent capability for MB adsorption.

### 3.5. Overall Sustainability Analysis

In recent years, public awareness of environmental protection has been increasing, and it is essential to conduct sustainability assessments. In order to evaluate the sustainability of the CIN/EP-ZrO_2_ composite membrane, we used the overall sustainability footprint (OSF) analysis by selecting eight factors, including the preparation simplicity, public acceptance, environmental friendliness, recyclability, degradability, removal efficiency, cost, and waste utilization rate, which were divided into low (L, *i* = 1), medium (M, *i* = 2), and high (H, *i* = 3) levels and calculated according to Equation (1).

**Figure 7:**
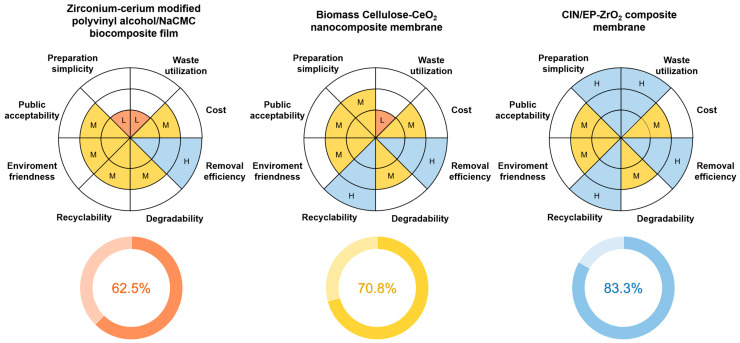
Comparison of the sustainability footprint of different biomass membranes.

Two membranes, Zirconium-cerium modified polyvinyl alcohol/NaCMC biocomposite membrane [56] and Biomass Cellulose–CeO_2_ nanocomposite membrane [57], were compared with the CIN/EP-ZrO_2_ composite membrane. As shown in Figure 7, the OSF value of Zirconium-cerium modified polyvinyl alcohol/NaCMC biocomposite membrane was 62.5%. The OSF value of the Biomass Cellulose–CeO_2_ nanocomposite membrane was 70.8%. The OSF value of our CIN/EP-ZrO_2_ composite membrane was 83.3%. Compared with both biomass composite membranes, the biomass composite membranes prepared in our study show great advantages in terms of simple preparation, recyclability, and waste utilization. Being a type of marine pollution, the resource utilization of EP has always been a concern to many. We choose EP as our raw material, which not only reduces the cost, but also realizes the resource utilization of EP. In addition, the CIN/EP-ZrO_2_ composite membrane prepared in this study shows a rapid and efficient adsorption ability for F^−^ and MB, as well as excellent antibacterial property, which proves that the CIN/EP-ZrO_2_ composite membrane has good sustainability and great potential in water treatment.

## 4. Conclusions

In summary, we reported the construction of a novel composite membrane for water purification applications. CIN was selected as a functional modifier combined with EP, and the CIN/EP-ZrO_2_ composite membranes were prepared by the biomimetic mineralization technique of ZrO_2_ NPs. Experimental results showed that the CIN/EP-ZrO_2_ composite membrane removed over 99.9% of F^−^ from water with concentrations of 100–400 mg/L. This high efficiency was achieved through the electrostatic interaction between the -OH groups on the surface of biomineralized ZrO_2_ NPs and F^−^ ions, and the formation of zirconium–fluoride complexes when F^−^ replaces the surface -OH groups. The composite membrane also significantly inhibited *E. coli* due to the synergistic effects of EP, CIN, and ZrO_2_. Additionally, it could adsorb 99.99% of MB from solutions with concentrations of 5–300 mg/L. This is mainly because the -OH, -COOH, and -NH_2_ groups in EP interact with MB molecules, giving the composite membrane an excellent adsorption capacity for MB. Furthermore, the sustainability of the CIN/EP-ZrO_2_ composite membrane was evaluated by OSF analysis, indicating that the CIN/EP-ZrO_2_ composite membrane had good sustainability and great potential in water purification. The CIN/EP-ZrO_2_ composite membranes prepared in this study have the advantages of simple preparation, low cost, and high adsorption performance. It is expected that the composite membranes can be widely used for the removal of ions, bacteria, and dyes from water to produce clean water with high efficiency.

## Figures and Tables

**Table 1:** Adsorption kinetic parameters of the CIN/EP-ZrO_2_ composite membrane towards F^−^.

Adsorbent	Pseudo-First-Order			Pseudo-Second-Order		
	*Q_e_* (mg/g)	*k*_1_	R^2^	*Q_e_* (mg/g)	*k*_2_	R^2^
	489.0815	0.01182	0.9907	579.0373	2.2790 × 10^−5^	0.9987

**Table 2:** Adsorption isotherm parameters of CIN/EP-ZrO_2_ composite membrane F^−^.

Isotherm	Langmuir			Freundlich		
	*q_m_* (mg/g)	*K_L_* (L/mg)	R^2^	*K_F_* (mg/g)	n	R^2^
	577.1421	0.06114	0.9955	108.5427	3.0867	0.9138

**Table 3:** Comparison of the maximum adsorption capacity of other adsorbents to F^−^ in the reported studies.

Adsorption Materials	Adsorption Elements	Amount of Adsorption	Ref.
Mn-Zr-Ethanol composite	F^−^	32.87 mg/g	[39]
Zr-CS/GO membrane	F^−^	29.0588 mg/g	[20]
Zirconium oxide/PAN nanofibers	F^−^	67.51 mg/g	[40]
Hierarchical zinc-zirconium oxide microspheres	F^−^	107.41 mg/g	[41]
Hydrous zirconium oxide-impregnated CS	F^−^	222.2 mg/g	[42]
Porous zirconium alginate beads adsorbent	F^−^	32.797 mg/g	[43]
Zirconium(IV) immobilized carboxymethyl cellulose/multiwalled carbon nanotubes gels	F^−^	36.657 mg/g	[44]
Graphene/Zr on Activated Carbon	F^−^	81.47 mg/g	[45]
Fe_3_O_4_-ZrO_2_ NPs	F^−^	158.6 mg g	[46]
CIN/EP-ZrO_2_ composite membrane	F^−^	577.1421 mg/g	This study

## Data Availability

The original contributions presented in this study are included in the article. Further inquiries can be directed to the corresponding author.
